# Risk perception as a motivational resource during the COVID-19 pandemic: the role of vaccination status and emerging variants

**DOI:** 10.1186/s12889-024-18020-z

**Published:** 2024-03-06

**Authors:** Joachim Waterschoot, Maarten Vansteenkiste, Vincent Yzerbyt, Sofie Morbée, Olivier Klein, Olivier Luminet, Mathias Schmitz, Pascaline Van Oost, Eveline Van Raemdonck, Marie Brisbois, Omer Van den Bergh

**Affiliations:** 1 Faculty of Psychology, Department of Developmental, Personality and Social Psychology, Ghent University, Henri Dunantlaan 2, B-9000 Ghent, Belgium; 2 Institute for Research in the Psychological Sciences, Université Catholique de Louvain, Louvain-la-Neuve, Belgium; 3 Center for Social and Cultural Psychology (CeSCuP), Faculty of Psychological Sciences and Education, Université libre de Bruxelles, Bruxelles, Belgium; 4 Fund for Scientific Research (FRS-FNRS), Brussels, Belgium; 5 Health Psychology, Faculty of Psychology and Educational Sciences, University of Leuven, Leuven, Belgium

**Keywords:** COVID-19, Risk perception, Vaccination, Motivation, Behavior

## Abstract

**Background:**

People’s perceived risk of being infected and having severe illness was conceived as a motivational source of adherence to behavioral measures during the COVID-19 crisis.

**Methods:**

We used online self-reported data, spanning 20 months of the COVID-19 crisis in Belgium (*n* = 221,791; 34.4% vaccinated; July 2020 - March 2022) to study the association between risk perception and motivation.

**Results:**

Both perceived infection probability and severity fluctuated across time as a function of the characteristics of emerging variants, with unvaccinated persons perceiving decreasingly less risk compared to vaccinated ones. Perceived severity (and not perceived probability) was the most critical predictor of autonomous motivation for adherence to health-protective measures, a pattern observed at both the between-day and between-person level among both vaccinated and unvaccinated individuals. An integrated process model further indicated that on days with higher hospitalization load, participants reported being more adherent because risk severity and autonomous motivation for adherence were more elevated on these days.

**Conclusions:**

These findings suggest that risk severity served as a critical and dynamic resource for adherence to behavioral measures because it fostered greater autonomous regulation.

**Supplementary Information:**

The online version contains supplementary material available at 10.1186/s12889-024-18020-z.

Motivation played a key role during the COVID-19 pandemic to predict individuals’ short- and long-term adherence to quite intrusive health-protective behavioral measures, such as wearing face masks, physical distancing, and accepting a vaccine. Of particular importance is that individuals are autonomously motivated, that is, they fully endorse or internalize the necessity of requested health behaviors [1, 2]. Therefore, the question arises which factors underlie autonomous motivation.

In the present study, we focus on the role of risk perception [3], which denotes both the estimated probability of being infected by the virus and the probability of experiencing severe symptoms (e.g., [4]). We had two major aims. First, we wanted to describe the dynamic evolution of risk perception as a function of different variants emerging throughout the pandemic and the individuals’ vaccination status. Second, we aimed to investigate whether risk perception predicts autonomous motivation and which element (infection probability or severity) is most predictive. We considered both the between-day and between-person level of analysis and investigated the moderating role of the prevailing virus variant and people’s vaccination status. Because experts, politicians, and media reported daily about infections and hospitalization rates, the present study can help to fine-tune risk communication during a pandemic to foster greater motivation and adherence to behavioral measures. This study is part of a long-term and large-scale population study that was initiated right after the outbreak of COVID-19 in Belgium and lasted more than 2 and a half years [5].

## The role of motivation for health behavior

During the COVID-19 crisis, policymakers faced several motivational challenges. To avoid a steep rise in infections and hospitalizations overloading healthcare services, it was of paramount importance that citizens adhered to behavioral measures to contain infections and were willing to accept vaccination. This means that people had to be motivated to do so [6]. Yet, not all types of motivation induction yield the same effects. From the perspective of Self-Determination Theory (SDT, [7, 8, 9]), autonomous motivation, which denotes a full and voluntary endorsement of the requested health behavior, is of critical importance. When individuals are autonomously motivated, they have internalized the value of the health behavior such that they voluntarily regulate their behavior. For instance, they adhere to corona-safe behaviors to protect vulnerable people, to avoid overburdening the health care sector or simply to stay healthy themselves. In contrast, in the case of controlled motivation, people experience internal (e.g., feelings of guilt) or external pressure (e.g., avoiding being criticized) to perform the health behavior, signaling that they have not or only partially internalized the importance of performing the health behavior. Finally, amotivation indicates that people lack the competencies or energy to engage in the recommended health behavior or minimize its importance [10]. Abundant research in the health domain, including smoking cessation [11], weight loss [12] and physical activity [13], has shown that autonomous motivation is a more reliable predictor of long-lasting behavior change than controlled motivation and amotivation [14].

The COVID-19 crisis created the opportunity to test some of the basic premises of SDT at a population level and over an extensive period. The available research points to three key findings [5]. First, the benefits of autonomous motivation emerge for different relevant health behaviors during the COVID-19 crisis, such as adherence to safety measures (e.g., wearing face masks, keeping distance, and time spent at home; [1, 15]) and the acceptance of the vaccine [2, 16]. Second, the benefits of autonomous motivation are not just short-lived but also relate to long-term desirable outcomes, including a lower likelihood of infection and the acceptance of a booster vaccine [17]. Third, the beneficial role of autonomous motivation was not only observed at the between-person but also at the between-day level. That is, day-to-day variability in the autonomous motivation of the population predicts corresponding day-to-day variation in critical epidemiological parameters (i.e., lower infection and hospitalization rates) several weeks later [18]. Overall, these findings confirm that the benefits of autonomous motivation are wide-ranging, long-lasting, and robust.

## Risk perception as a motivational resource

The key aim of the present study was to examine the role of risk perception as a critical driver for autonomous motivation. Within different models of health behavior, such as the health belief model [4] and the health action process approach [19], risk perception refers to the judgments and assessments of an individual about potential threats and their short and long-term consequences. Applied to the COVID-19 context [20], risk perception involves two aspects, that is, the probability of being infected by the virus (i.e., personal vulnerability, infection probability) and the perceived severity of the symptoms after actual infection (i.e., severity). Both aspects can be estimated with respect to one’s personal situation and the population at large. Importantly, *perceived* risk can be different from *actual objective* risk [21, 22].

Risk perception has generally been found to be an important predictor of health behavior [23, 24], with action planning and coping planning as intermediate steps needed to guarantee that perceived risk translates into actual behavior [19]. In the present study, we aimed to systematically investigate the link between risk perception, autonomous motivation, and adherence to behavioral measures during the COVID-19 pandemic. We assumed that high perceived risks would provide a legitimate reason for people to endorse the importance (i.e., to internalize) of prescribed health-protective behaviors. Wise et al. [24] provided some preliminary evidence for this assumption by showing that individuals who perceived higher risks for themselves or for the population also found it more valuable and meaningful to conform to the recommended safety measures during the pandemic. A similar pattern of findings was observed for vaccine uptake. Individuals who anticipated becoming severely ill after infection reported higher autonomous motivation for vaccination uptake, which eventually predicted a higher effective uptake of the vaccine [2].

The present study builds on this limited evidence by examining the unique and potentially different role of both aspects of risk perception as resources of autonomous motivation. Prior research indicates that perceived severity typically yields stronger associations with health behaviors than the perceived probability of infection (e.g., [20, 25, 26]). Along similar lines, the severity aspect may play a more decisive role in fostering autonomous motivation for health-protective behaviors than the perceived probability of infection. In addition, we also considered the role of people’s vaccination status and the type of circulating virus as potential moderators of the association between risk perception and autonomous motivation.

## Role of the evolution of the crisis and vaccination status

Throughout the COVID-19 crisis, different virus variants became dominant at different moments in time. Because some variants were more contagious, yet less ill-making than others, changes in people’s risk perception may be noticed accordingly. Only a limited number of previous studies focused on context-dependent variation of risk perception within persons. Wise et al. [24] reported longitudinal increases in perceived risk during a period of increasing infection numbers, whereas Chen et al. [27] reported lower perceived risk after the introduction of lockdown measures. Also, levels of risk perception decreased as the crisis unfolded, presumably because people perceived the virus to be less unfamiliar and less unpredictable [28]. To illustrate, the emergence of the Omicron variant changed the trajectory of the COVID-19 pandemic [29]. Although its high contagiousness was a serious concern, people became also more optimistic as Omicron was less ill-making than previous variants [30, 31]. Yet, the literature remains scarce and no long-term studies are available so far that describe evolutions in risk perception as a function of different virus variants. Also, the question of whether the association between risk perception and autonomous motivation varies across different phases has not been addressed.

Not only the dominant virus type, but also the relative number of vaccinated and unvaccinated individuals changed across time, which may have impacted the motivational profile of the assessed persons. Initially (in the spring and summer of 2021), the group of unvaccinated persons was large and comprised a mix of uninvited, doubting, and refusing individuals. However, this heterogeneity may have decreased as the vaccination campaign progressed: the majority of people had received a vaccine by the end of 2021, leaving only strongly resistant individuals unvaccinated.

Prior studies reporting a positive association between risk perception and vaccination intention [2] were conducted before or at the beginning of the vaccination campaign. Only a few studies showed a difference in risk perception between those already being vaccinated and unvaccinated [22]. Therefore, we aim to examine mean-level differences between vaccinated and unvaccinated persons as regards both aspects of risk perception, autonomous motivation, and behavioral adherence.

## The present study

**Fig. 1:**
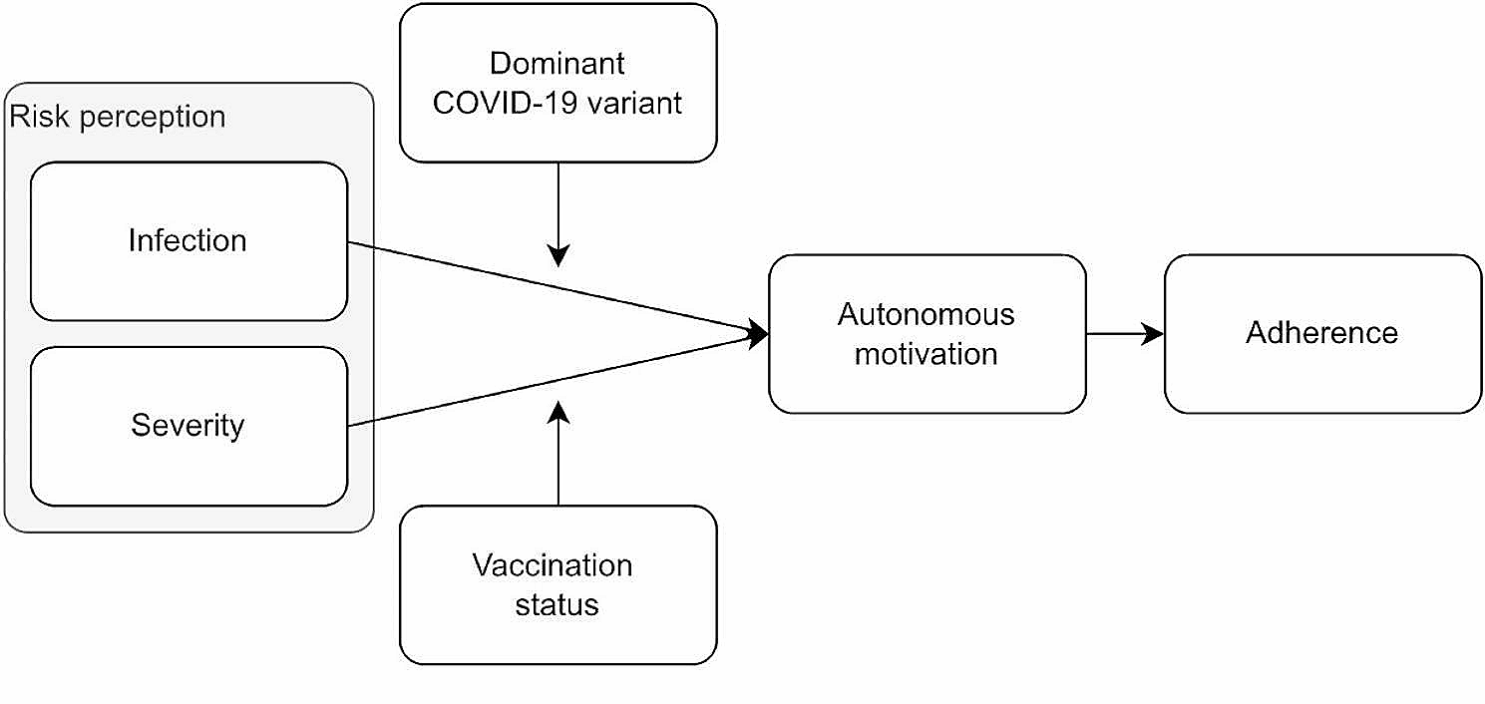
Examined conceptual model

We investigated the evolution and the role of risk perception as a predictor of autonomous motivation or volitional internalization of health-protective behaviors throughout the pandemic. Figure 1 provides an overview of the conceptual model. We monitored citizens’ risk perception (i.e., both perceived probability and severity), autonomous motivation, and behavioral adherence during 20 months of the COVID-19 pandemic starting in July 2020.

Our large dataset allowed us to pursue two aims. First, we wanted to describe the dynamic evolution of risk perception as a function of the dominant virus variant circulating in society and of people’s vaccination status. Although we expected both aspects of risk perception to evolve in parallel throughout most of the pandemic, we predicted that the emergence of the highly contagious, yet less severe Omicron variant in the fall of 2021 may have led to a dissociation with an increasing perceived infection probability and a decreasing perceived severity of illness [29] (Hypothesis 1a). Further, we hypothesized that unvaccinated persons would report fewer risks compared to vaccinated persons, with the average discrepancy between both groups widening across time as both groups become increasingly more homogeneous (Hypothesis 1b).

Second, at a structural level, we examined the role of risk perception as a predictor of autonomous motivation. We hypothesized that perceived severity would be a better predictor than perceived probability for the internalization and the willingness to adhere to health-protective measures (Hypothesis 2). In a more exploratory way, we examined whether the risk-motivation association depended on the specific variant (i.e., Omicron) and the individuals’ vaccination status. One possibility is that unvaccinated persons may not only perceive fewer risks, but also be less responsive to the risk perception-motivation link. Similarly, as the Omicron variant was accompanied by less severity, the motivating potential of this aspect of risk may decrease as well.

Finally, given that we possessed daily assessments of risk perception, motivation, and adherence and could retrieve objectively recorded daily infections and hospitalizations, we sought to examine the sequential model depicted in Fig. 1 (Hypothesis 3). We hypothesized that participants would report a higher risk for infection on days with higher numbers of infections, while a higher risk for severe illness was expected on days with a higher number of hospitalizations. Subsequently, we expected that daily variation in risk perception would serve as an intermediate mechanism between daily variation in these objective parameters and daily variation in autonomous motivation, which, in turn, would predict daily variation in behavioral adherence.

## Methods

### Participants and procedure

The current data collection took place in the context of a nationwide research project called ‘the Motivation Barometer’ in Belgium [5]. Through an online questionnaire, which we constructed in Qualtrics, the project monitored various aspects of people’s psychological functioning, including their well-being, risk perception, motivation, and adherence to the COVID-19 measures. We distributed the survey online through organizations, national newspapers (e.g., Het Nieuwsblad, Le Soir, etc.), social media (Facebook, Instagram, Twitter, Linkedin), and our website (www.motivationbarometer.com) and promoted it by advertisements in which the topic was briefly explained. After opening the survey and reading the introduction about the goals of the research project, participants had to complete an informed consent explaining that their participation was voluntarily, that data would be analyzed anonymously, and that they could end their participation at any time without consequences. In addition, we provided contact information in case of questions or negative feelings. The project was approved by the ethical committee of Ghent University (N° 2020/37). We did not pre-register the study and its hypotheses because the current study and its research questions developed dynamically during the pandemic depending on the changing circumstances, which did not allow us to pre-register the hypotheses.

The study had a serial cross-sectional design and took place between July 2020 and March 2022. Across 593 days (61% with *n* > 40), 221,791 unique participants completed the survey (*M*_*age*_ = 47.82, range = 18–82; 61.8% female; 34.4% vaccinated participants; 64.4% of all participants having started the survey; see Figure S1 for an overview). From this sample, 80.3% reported no comorbidity condition, 16.4% reported one, and 3.3% reported to have more than one comorbidity. In terms of education, 33.1% reported to have no graduation or one in secondary school, 38% reported a Bachelor’s degree and 28.9% reported to have a Master’s degree.

### Measures

Prior to measuring the psychological variables, we asked for people’s age, gender (i.e., male or female), vaccination status (i.e., vaccinated or not vaccinated), number of comorbidities (i.e., respiratory condition, diabetes, heart disease or hypertension, lung disease, liver disease, cancer, disease affecting the immune system, and a disease not specified in this list), and education level (i.e., no graduation or secondary graduation, Bachelor degree or Master degree).

#### Risk perception

We measured risk perception using four items [32]. Two items assessed participants’ estimated probability to be infected by the coronavirus in the near future (1 = ‘Very small’ to 5 = ‘Very big’; *r*_between−person_ =.43, *r*_between−days_ =.87, *p’s <.001*) and two items assessed participants’ estimated severity of the symptoms when being infected (1 = ‘Not at all serious' to 5 = ‘Very serious’; *r*_between−person_ =.62, *r*_between−days_ =.84, *p’s <.001*). They answered both questions twice, once with respect to themselves and once with respect to the Belgian population.

#### Autonomous motivation

We assessed people’s motivation to adhere to the corona safety measures with an adapted version of the Behavioral Regulation in Sport Questionnaire [33]. After the stem “Over the past week, I’ve adhered to these measures because”, people answered four items for autonomous motivation on a 5-point scale ranging from 1 (“not at all true”) to 5 (“totally true”). Two item examples are “because I find it personally relevant” and “because these behaviors are an expression of my personal values”. (*α*_between−person_ = .89; *α*_between−days_ = 0.92).

#### Adherence to the measures

We tapped people’s self-reported adherence with one item for each of the three most important and stable COVID-19 measures introduced in Belgium that were put in place across the assessment period, that is, “to wash your hands frequently”, “to wear your face mask when mandatory or recommended”, and “to maintain physical distance from others.” Participants were asked to indicate on a scale ranging from 1 (“I do not adhere to it at all”) to 5 (“I totally adhere to it”) the extent to which they followed each of the three measures (*α*_between−person_ = .72; *α*_between−days_ = .92).

#### Epidemiological data

We obtained data on daily infections and daily hospitalizations from Sciensano, the national public health institute [34]. As these parameters are expressed in exponentials, we applied a log transformation to include these variables in linear analyses.

### Phases of the pandemic

**Fig. 2:**
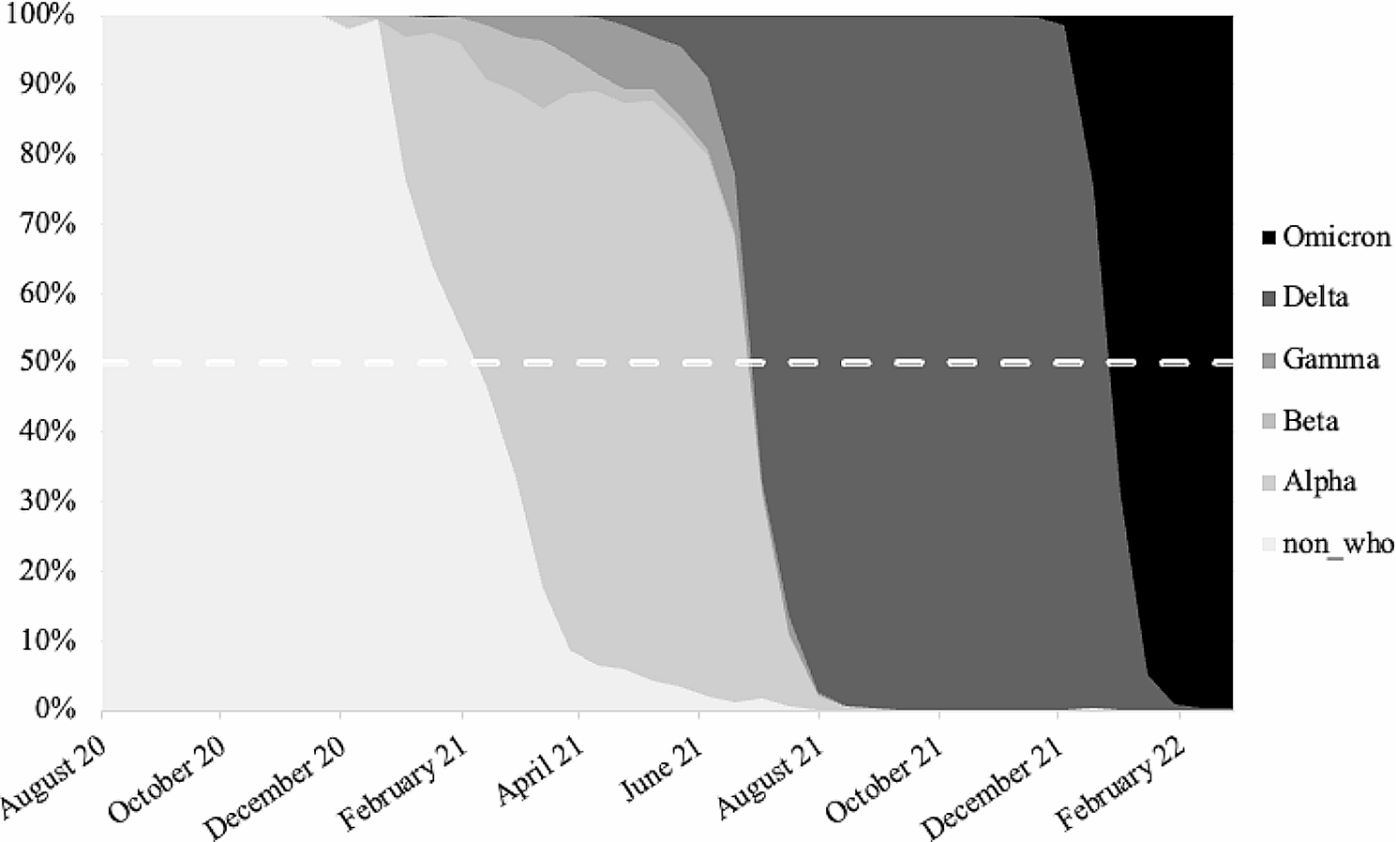
Overview of percentual shift of different COVID-19 variants in Belgium across the pandemic. *Note*. non_who represents those variants that were unlabeled by the World Health Organization (WHO)

We divided the total period into distinct phases based on the dominance of a SARS-CoV-2 variant, with dominance being reached when more than 50% of the identified cases on a given day is caused by the same variant (e.g., [35, 36, 37]). As can be noticed in Fig. 2, four different phases were distinguished. As no single variant was dominant in the first phase of the pandemic, this phase was labeled as *Undefined* (i.e., 10 July 2020–20 February 2021). From February 2021 onwards, the *Alpha* (i.e., 21 February– 30 June 2021), *Delta* (i.e., 1 July– 29 December 2021), and *Omicron* (i.e., 30 December 2021–3 March 2022) variant became consecutively dominant, with these three phases being labeled accordingly.

### Analysis plan

Before conducting the preliminary and main analyses, we focused on missing data analysis, thereby reporting the percentage of missing values for each study variable (see Table 1) [38]. Because more than 10% of the sample is composed of partial respondents (i.e., reporting at least one construct but also missing at least one construct), the multiple imputation approach (i.e., 50 times in the current study) using the predictive mean matching algorithm [39] was used as the missing data treatment in the current study [40]. Doing this has the advantage of generating unbiased and accurate standard errors under MCAR (Little’s [41] MCAR test: χ^2^ (17) = 16.03, *p* =.62), which is appropriate for hypothesis testing [42]. In doing this, we included all study variables, including missing data, as well as sociodemographic and crisis-related variables (i.e., cases, hospitalizations). All subsequent analyses and reported results are based on this (pooled) imputed dataset.

**Table 1:** Descriptive statistics and Pearson correlations between assessed variables at the between-day level (below the diagonal) and the between-person level (above the diagonal)

Variable	M	SD	ICC		1.	2.	3.	4.	5.
1. Perceived infection	2.97	0.84	.09			.46^***^	.38^***^	.28^***^	
2. Perceived severity	2.93	0.93	.15		.03		.61^***^	.50^***^	
3. Autonomous motivation	3.36	1.22	.12		.12^**^	.76^***^		.65^***^	
4. Adherence	3.90	0.99	.11		.04	.82^***^	.75^***^		
5. Infections	8.26	1.21	-		.63^***^	−.38^***^	.03	.01	
6. Hospitalizations	7.61	0.68	-		.29^***^	.08	.09^**^	.05^*^	.41^***^

Note. * p <.05, ** p <.01, ***p <.001. Perceived infection and perceived severity had, respectively, 14% and 15% missing values, while autonomous motivation and adherence contained, respectively, 4% and 5% missing values

A set of preliminary analyses was conducted to check for the effect of sociodemographic variables on the study variables. Categorical background variables (i.e., gender, comorbidity, education level) were examined using a MANCOVA to identify multivariate effects, and through ANOVA’s to identify univariate effects. Pearson correlations were used to examine the associations between age and assessed outcomes at the between-subject level. Indeed, to examine the associations between the (continuous) study variables, we calculated Pearson correlations on both the between-subjects and the between-days levels. The use of such a multilevel approach was justified by the Intra-Class Coefficient (ICC), representing the similarity of participants within days or, put differently, the variation between days.

To examine mean level changes in aspects of risk perception, autonomous motivation, and behavioral adherence across different phases of the pandemic as a function of vaccination status (Hypothesis 1), we performed a series of multiple linear mixed regression models. Herein, the variable *days* was included as a random intercept, because accounting for a meaningful amount of between-days variance (see ICC-values) avoids biased parameter estimates [43]. Within these models, the phasing of the pandemic is introduced as a predictor at the between-day level, while vaccination status and background variables are introduced as predictors at the between-person level. A cross-level interaction, testing for the interplay between phasing and vaccination status, was introduced as an additional predictor. In these analyses, the first phase (i.e., *Undefined*) was not included as the vaccination rollout had not started yet at the time, thus preventing us from examining the role of vaccination status. Further, we centered continuous variables in the models and checked model assumptions (i.e., normality of residuals, influential observations). Multicollinearity was checked by the Variance Inflation Factor (VIF > 4 indicates multicollinearity). The output of the model involves the ANOVA reporting of the F-values, *p*-values for the statistical significance and partial eta-squares (*η*^*2*^_*p*_) as effect sizes for the sake of practical significance [44]. With the current large dataset, *p-*values tend to become significant when effects are small. Effects are interpreted as small when *η*^*2*^_*p*_ *>* 0.0099, medium when *η*^*2*^_*p*_ *>* 0.0588 and large when *η*^*2*^_*p*_ *>* 0.1379 ([45]; pp. 278–280). In the visual output of the model, we still included the average of the *Undefined* phase as it serves as a useful reference point in the crisis.

To examine the structural role of risk perception in the prediction of autonomous motivation and adherence (i.e., Hypothesis 2), we used a similar procedure. We introduced phases at the between-day level and risk perception and aspect of risk perception (i.e., probability, severity) at the between-person level. Also, the cross-level interaction between all three predictors was inserted to examine whether the descriptive evolution of risk perception was phase- and aspect-dependent. In an additional set of analyses, we investigated whether the evolutions in risk perception across time would depend on vaccination status (thus involving a 4-way interaction between phase, risk perception, aspect of risk perception and vaccination status). These analyses were performed on a truncated sample, that is, a sample excluding the first phase of the pandemic because all individuals were still unvaccinated at that time.

To examine the extent to which daily risk perception would serve as a resource of daily autonomous motivation and subsequent daily adherence, while being predicted by daily variation in the epidemiological situation (i.e., Hypothesis 3), we performed a Multilevel Structural Equation Model (MSEM) using the R-package lavaan [46]. We created latent variables for both aspects of risk perception, autonomous motivation, and adherence. This model was examined at the between-day level, while controlling for between-person differences in the variables in the model.

We report how we determined our sample size, all data exclusions (if any), and all sanitary behaviors in the study, and we follow JARS. All data, analysis code, and research materials are available at Zenodo (after contacting the responsible author) and Open Science Framework (https://osf.io/5cqhr/). We analyzed the data using R, version 4.1.2.

## Results

### Preliminary analyses

As can be noticed in Table 1, ICC values were lower than 0.15, indicating that most of the variance is located at the between-person (i.e., or within-days) level. At the same time, a significant amount of variance in all outcomes varied between days. Therefore, we computed Pearson correlations on both the between-person (i.e., upper triangle) and the between-day levels (i.e., lower triangle). All variables were positively correlated at the between-person level. This pattern was largely mirrored at the between-day level, with the exception that daily perceived infection was unrelated to daily perceived severity and behavior and only minimally related to daily autonomous motivation.

Further, daily variation in registered infections was related positively to daily perceived infection and negatively to daily perceived severity. No correlation was found with autonomous motivation and adherence. Further, daily hospitalizations are related positively to daily perceived infection, autonomous motivation and adherence.

Further, MANOVA analyses provided evidence for significant multivariate effects for gender (Wilk’s lambda = 0.97, *F*(4, 127,998) = 947.22, *p* <.001), comorbidity (Wilk’s lambda = 0.94, *F*(8, 256,862) = 1033.79, *p* <.001) and education level (Wilk’s lambda = 0.97, *F*(8, 246,602) = 442.01, *p* <.001). Table S1 shows univariate analyses with effect sizes, indicating that females and individuals with comorbidities scored higher on both aspects of risk perception, autonomous motivation, and adherence. Effect sizes were small. Next, age was positively related to perceived severity (*r* =.29, *p* <.001), autonomous motivation (*r* =.24, *p* <.001) and adherence (*r* =.20, *p* <.001), while being unrelated with perceived risk (*r* =.02, *p* =.10).

### Hypothesis 1: mean level differences

**Fig. 3:**
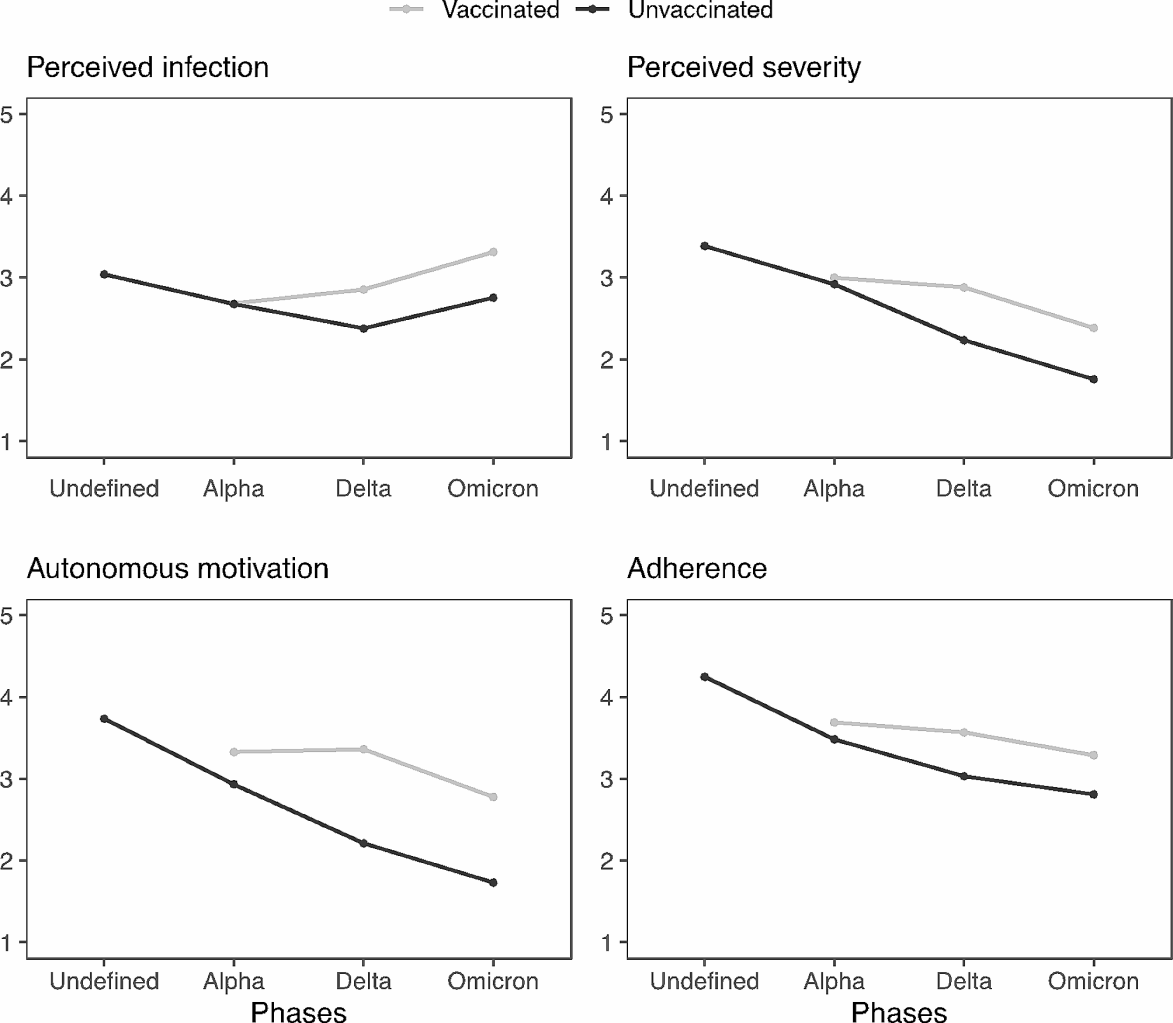
Visualization of two-way interactions between phases and vaccination status. *Note*. The mean of the *Undefined* phase was added to this figure for the sake of information. Values from the *Alpha* to the *Omicron* phase are the output of the model in Table 2

The ANOVA output of the linear mixed regression models in Table 2 provides evidence for a main effect of phase and vaccination status, with cross-level interaction effects with a small to medium effect size also reaching significance in the prediction of both aspects of risk perception, autonomous motivation, and adherence. Also, no model assumptions were violated and no multicollinearity was detected. The significant two-way interaction effects are visualized in Fig. 3, thereby including the means in phase *Undefined* as a reference point for comparison purposes. First, the main effect of phases indicates that there was a steady decrease in perceived infection and perceived severity, autonomous motivation, and adherence across the different phases, except for perceived infection which increased again in the *Omicron* phase. Second, the significant phase by vaccination status interaction suggests that the difference between vaccinated and unvaccinated persons widened in the transition from the *Alpha* to the *Delta* phase and remained rather stable during the subsequent *Omicron* phase. Specifically, vaccinated persons reported higher risk perception for both aspects, higher autonomous motivation and higher adherence from the *Delta* phase on compared to the unvaccinated.

**Table 2:** ANOVA output of linear mixed regression model including *F*-values and partial eta-squared values

	Perceived infection					Perceived severity					Autonomous motivation					Adherence			
**Fixed effects**	F-value	*p*-value		*η* ^*2*^_*p*_		F-value	*p*-value		*η* ^*2*^_*p*_		F-value	*p*-value		*η* ^*2*^_*p*_		F-value	*p*-value		*η* ^*2*^_*p*_
***Between-subject predictors***																			
Age	760.38	<.001^***^		.01		3536.33	<.001^***^		.04		2723.83	<.001^***^		.04		2113.59	<.001^***^		.03
Gender	736.18	<.001^***^		.01		2147.62	<.001^***^		.02		1624.76	<.001^***^		.02		3142.29	<.001^***^		.04
Comorbity	247.74	<.001^***^		.00		2398.83	<.001^***^		.02		457.23	<.001^***^		.00		303.89	<.001^***^		.00
Education level	111.76	<.001^***^		.00		149.16	<.001^***^		.00		17.05	<.001^***^		.00		29.91	<.001^***^		.01
Vaccination status	2868.68	<.001^***^		.15		4935.84	<.001^***^		.10		6115.60	<.001^***^		.14		1631.67	<.001^***^		.13
***Between-days predictors***																			
Phases	23.24	<.001^***^		.03		437.53	<.001^***^		.05		129.54	<.001^***^		.04		93.06	<.001^***^		.02
***Cross-level interaction***																			
Phases : Vaccination status	526.01	<.001^***^		.04		536.83	<.001^***^		.06		575.03	<.001^***^		.05		88.41	<.001^***^		.03
**Random effects**:																			
Number of days	0.22					0.14					0.22					0.14			
Residual	0.81					0.78					1.11					0.99			
Max VIF	3.05					3.07					2.94					3.02			
*R*^*2*^ (marginal/conditional)	.09/.15					.26/.27					.19/.22					.11/.13			

Note. ^***^p <.001, ^**^p <.01, ^*^p <.05

### Hypothesis 2: predictive validity of risk perception

**Fig. 4:**
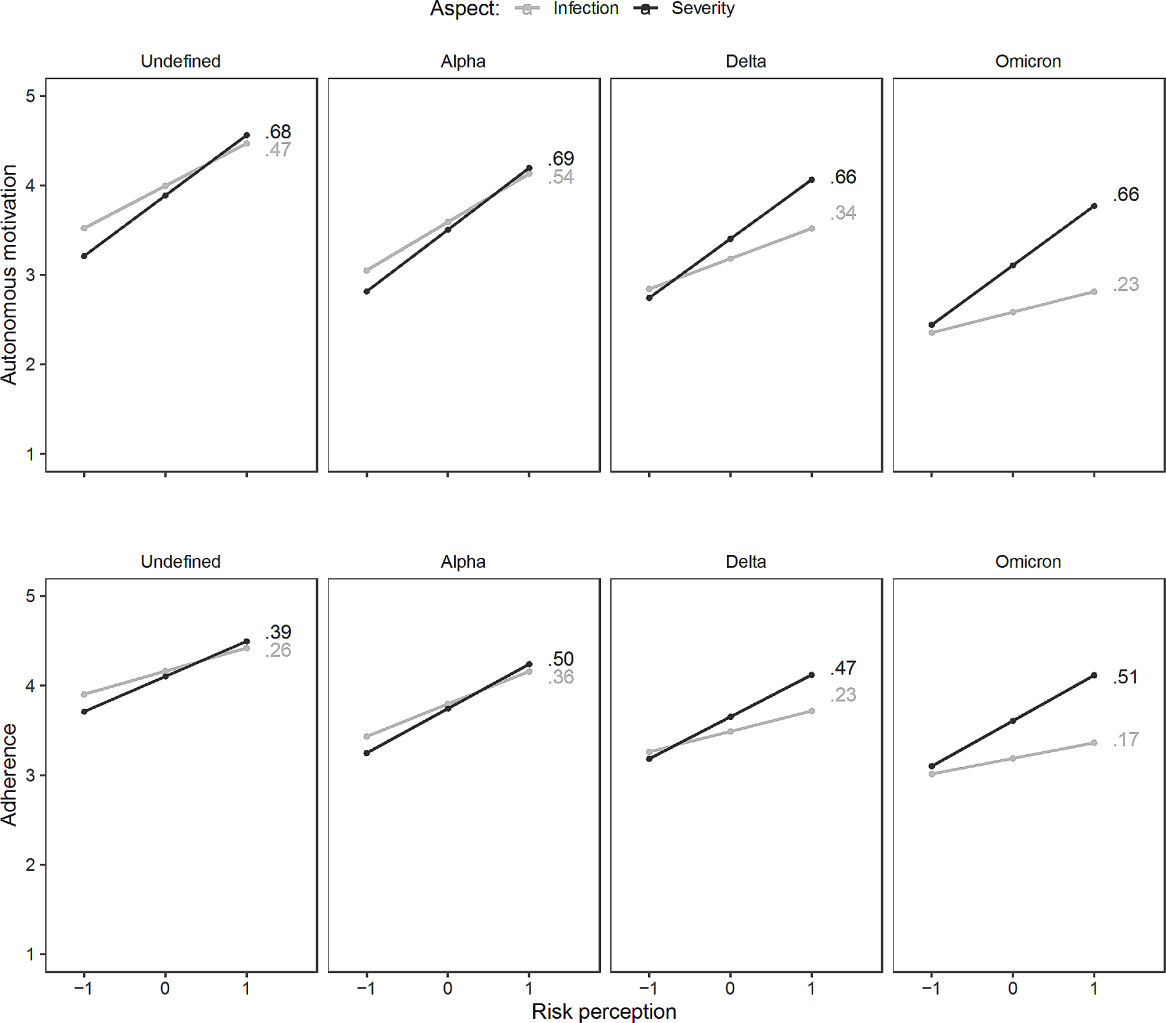
Visualization of three-way interactions between dominant variant and aspect of risk perception in the prediction of autonomous motivation and adherence with standardized simple slope coefficients. *Note*. All standardized simple slope coefficients were significant with *p* <.01

To examine the predictive validity of both aspects of risk perception, we first performed linear mixed regression models including a three-way interaction between risk perception, the aspect of risk perception, and phase. As can be noticed in Table 3, a risk perception by aspect interaction of medium effect size was obtained. Figure 4 shows that perceived severity was more strongly associated with both autonomous motivation and adherence than perceived probability. This significant two-way interaction was further moderated by the phase of the pandemic, thus yielding a significant three-way interaction with a small effect size. As can be seen in Fig. 4, the relation of perceived infection with autonomous motivation (grey lines; upper figure) and behavioral adherence (bottom figure) decreased across time, while associations between perceived severity and both autonomous motivation and adherence (black lines) remained stable across the different phases of the pandemic. Specifically, the most pronounced differences in the role of both aspects of risk perception are observed in the *Omicron* phase, during which perceived probability of infection was more modestly related to both outcomes compared to the previous phases.

**Table 3:** output of linear mixed regression model including standardized coefficients and partial eta-squared values

	Autonomous motivation					Adherence			
**Fixed effects**	β	*p*-value		*η* ^*2*^_*p*_		β	*p*-value		*η* ^*2*^_*p*_
***Between-subject predictors***									
Age	.13	<.001^***^		.02		.13	<.001^***^		.02
Gender[female]	.07	<.001^***^		.01		.13	<.001^***^		.02
Comorbity	.02	<.001^***^		.00		−.02	<.001^***^		.00
Education level	.03	<.001^***^		.00		.03	<.001^***^		.00
Vaccination status[unvaccinated]	−.25	<.001^***^		.03		−.11	<.001^***^		.01
Aspect[severity]	−.04	<.001^***^		.04		−.03	<.001^***^		.00
Risk perception	.37	<.001^***^		.15		.25	<.001^***^		.09
Aspect[severity] : Risk perception	.11	<.001^***^		.06		.09	<.001^***^		.05
***Between-days predictors***									
Phases[Alpha]	−.14	<.001^***^		.04		−.15	<.001^***^		.03
Phases[Delta]	−.30	<.001^***^				−.30	<.001^***^		
Phases[Omicron]	−.32	<.001^***^				−.27	<.001^***^		
***Cross-level interactions***									
Phases[Alpha] : Aspect[severity]	.01	<.001^***^		.01		.00	.21		.01
Phases[Delta] : Aspect[severity]	.10	<.001^***^				.08	<.001^***^		
Phases[Omicron] : Aspect[severity]	.11	<.001^***^				.10	<.001^***^		
Phases[Alpha] : Risk perception	.02	<.001^***^		.02		.05	<.001^***^		.01
Phases[Delta]: Risk perception	−.06	<.001^***^				−.02	<.001^***^		
Phases[Omicron]: Risk perception	−.06	<.001^***^				−.03	<.001^***^		
Phases[Alpha] : Aspect[severity]: Risk perception	−.01	<.001^***^		.03		.00	.11		.02
Phases[Delta] : Aspect[severity]: Risk perception	.04	<.001^***^				.04	<.001^***^		
Phases[Omicron] : Aspect[severity]: Risk perception	.04	<.001^***^				.04	<.001^***^		
**Random effects**:									
Number of days	0.14					0.17			
Residual	0.83					0.87			
Max VIF	3.56					3.76			
*R*^*2*^(marginal/conditional)	.35/.41					.29/.35			

Note. ^***^p <.001, ^**^p <.01, ^*^p <.05

In a next step, we checked whether the pattern of associations between both aspects of risk perception and outcomes across the pandemic would be any different for vaccinated versus unvaccinated persons. The effect sizes of these four-way interactions were zero, indicating that the associations observed in Fig. 4 equally apply to vaccinated and unvaccinated persons.

### Hypothesis 3: risk perception as an intervening mechanism

**Fig. 5:**
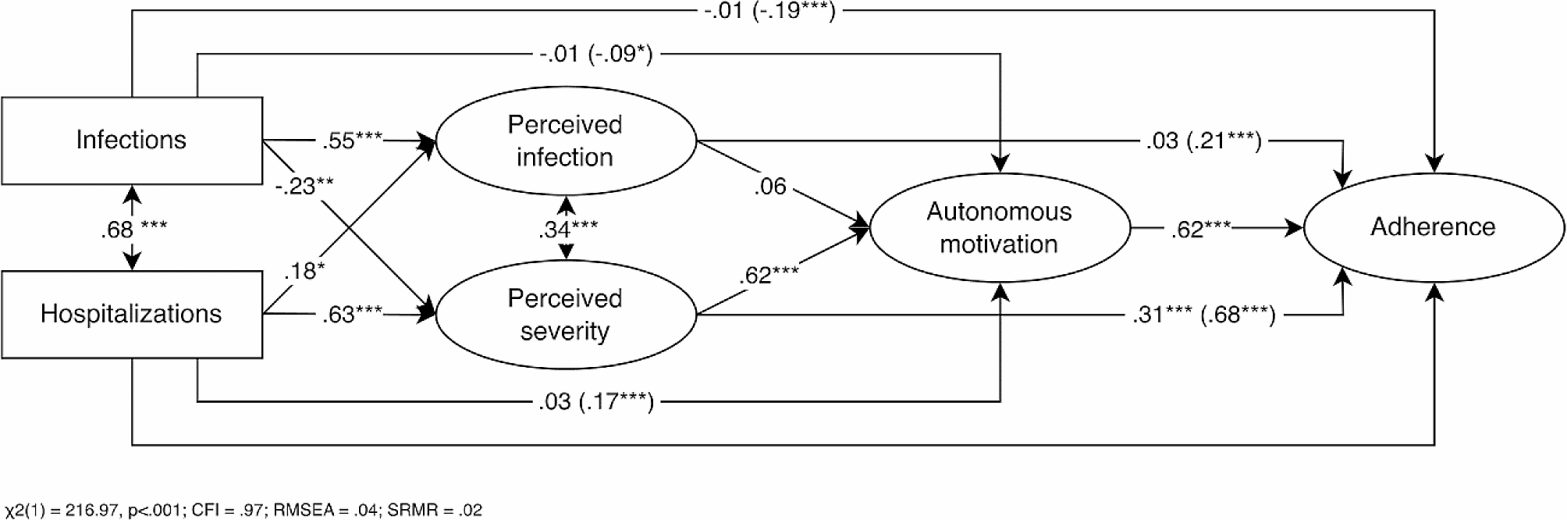
Visualization of a multilevel structural equation model with standardized coefficients reflecting between-day associations. *Note*. ^***^p <.001, ^**^p <.01, ^*^p <.05

Figure 5 shows the output and fit indices of the M-SEM with standardized coefficients on the between-day level. Daily variation in infection and hospitalization numbers related positively with, respectively, perceived infection probability and perceived severity. Daily perceived severity (and not perceived probability), in turn, related positively to daily autonomous motivation, which related positively to daily adherence. A significant indirect effect (β_indirect_ = .24, *p* <.001) was obtained from daily hospitalizations to daily adherence via daily levels of perceived severity and autonomous motivation. Such a pathway was not found for perceived infection by infection numbers (β_indirect_ = .02, *p* =.43). Finally, it is worth noting that daily hospitalizations still yielded a supplementary positive association with daily autonomous motivation and that daily perceived severity yielded a supplementary positive association with daily adherence to health-protective measures.

## Discussion

The present study provides a unique and fine-grained insight into the evolution and role of risk perception during the COVID-19 pandemic. Prior research indicated large individual differences in people’s perceived risks, which explain variability in one’s (intentions to) adherence to the sanitary measures [19], autonomous motivation for vaccination [2] and intention to take the vaccine [17]. We aimed to extend this body of work by monitoring individuals’ perceived risk for 20 months (i.e., descriptive aim) and examining which aspect of risk perception (i.e., probability of infection or severity) yielded the strongest motivational effect (i.e., structural aim), an issue we explored at both the between-person and between-day level. In addressing both aims, we also considered the role of vaccination status and the phase of the pandemic. The data allowed us to highlight four key findings.

First, the perceived severity of symptoms after infection was found to be a more salient aspect of risk than the perceived probability of becoming infected throughout the pandemic until Omicron emerged. At that point, the more contagious, yet less sick-making character of Omicron altered the individuals’ risk perception and both aspects of risk started to dissociate rather than evolving in parallel.

Second, individuals’ vaccination status reduced the perceived risk, a finding in line with earlier literature [3]. Yet, the time frame of the present study allowed us to observe a widening difference between the vaccinated and unvaccinated persons across time. Presumably, in the early months of the vaccination campaign, unvaccinated individuals represented a more heterogeneous group, with the groups becoming increasingly homogeneous as the pandemic evolved. Likely, the unvaccinated group gradually consisted of people who explicitly refused the vaccine. Despite being better protected against illness, the vaccinated people still perceived higher risks than unvaccinated, a finding also reported by Qin et al. [3]. Several reasons may help to account for these mean-level differences. Unvaccinated persons may adjust their behavior to minimize risks (e.g., avoiding close contacts), they might follow different types of media than vaccinated persons do or they may minimize the risks to justify their lack of vaccination. Future longitudinal research could follow-up the same participants to examine whether such a decrease effectively emerges across time within the same group of participants.

Third, as hypothesized, only the severity aspect of risk was related to autonomous motivation to adhere to the measures. As can be noticed in Fig. 4, each of the associations between perceived severity and autonomous motivation was significant and positive across the four phases of the pandemic, a finding that applied to both vaccinated and unvaccinated individuals. Despite the mean level differences in risk perception between both groups, perceived risk was found to have a similar motivating role for both. Stated differently, both groups seem equally risk responsive, although the threshold to perceive risks differed between both groups.

Fourth, the critical role of severity was also documented at the between-day level. That is, on days when the population reported higher risks, they were more autonomously motivated, which in turn led to higher adherence to the prevailing corona measures at that moment. Interestingly, objectively registered hospitalizations on a given day were positively related to perceived severity, but were unrelated to perceived infection probability. In turn, infection numbers were related to perceived infection probability, but did not act as a predictor of autonomous motivation on a daily base.

In terms of theoretical relevance, our findings shed light on an underexplored source of internalization within Organismic Integration Theory [47, 33], one of the six mini-theories of Self-Determination Theory. For people to fully accept and internalize a behavioral regulation, they need to perceive the behavior as meaningful and valuable. When the risk of severe illness, either for oneself or for close others, was elevated, people perceived engagement in health behaviors as more valuable and important. Perceived severity, therefore, might provide a legitimate reason for people to engage in long-lasting health-protective behavior. This finding is intriguing and particularly important because strong internalization of health behaviors was observed despite the long lasting violation of the basic needs of people for autonomy, competence, and relatedness [48]. For example, keeping physical distance runs counter to our natural tendencies and may be experienced as socially alienating. Despite these frustrations of basic needs, risk severity served as an important source of internalization. Presumably, risk severity was a legitimate and sufficient reason to endorse health-protective behaviors. At the same time, risk severity seems a rather fragile source of internalization, with its motivational power quickly fading out when risks were no longer perceived as severe.

### Practical implications

The current study provides novel insights into the motivational effects of risk perception and its relevance for communication strategies during crisis management. First, the consistent role of autonomous motivation in predicting health protective behavior underscores its practical importance. People are not just carrying out (or oppose) instructions by policymakers. They are problem solvers themselves who want to understand the problem and the ways to contain it. This means that policymakers should address them as co-problem solvers together with the policymakers and should use any means to induce this sense of co-responsibility rather than putting pressure on people to act responsibly. Presenting graphical ‘if-then’ scenarios could provide such information where prospective infection curves are related to different levels of behavioral adherence [49].

Second, perceived severity appears a more powerful determinant of autonomous motivation for health protective behavior than perceived probability. Interestingly, actual hospitalization rates as communicated by the media impact particularly perceived severity and, as such, already contributes to internalized motivation. However, several caveats should be added here. For example, the dissociating association of perceived infection probability and perceived severity with autonomous motivation observed during the Omicron variant [50] suggests the need for continuous monitoring of risk perception and flexible strategies to adapt crisis communication appropriately. Another caveat is that enhancing perceived severity in risk communication may induce worry and anxiety in a substantial part of the population (e.g., [51]). The implication is that strategies to enhance severity perception should be flanked by clear communication of behavioral options to empower people to efficiently avoid or reduce the perceived severity (among which is, for example, vaccination).

Third, individual differences and contextual variables play an important role in the divergence between perceived risk and actual risk, as is illustrated by our findings on the role of vaccination status. Despite being less protected against illness, unvaccinated persons perceived lower risks than vaccinated persons, which probably contributed to the decision not to take a vaccine. Unvaccinated persons may need tailored information. Our findings suggest that personalized severity information could contribute to foster autonomous motivation to take a vaccine. Fourth, we believe that our findings are not only critical for managing a pandemic, but also offer valuable lessons for future societal challenges that require behavioral involvement of the population, such as countering climate change [5].

### Limitations

The current research has several limitations that help to contextualize the findings. First, because of its self-selected nature, the current sample was not representative of the Belgian population. Specifically, the current sample is older than the average population (*M*_*age*_ = 41.4) and include more female participants (51%), which may have led to an overestimation of risk and autonomous motivation across the pandemic as older and female participants on average scored higher on these outcomes. Some argued for alternative methods to deal with non-representativity of datasets, like using weighing. However, such a procedure might result in biased parameters and loss of accuracy [52], while the usefulness of weighting in datasets may be less relevant when focusing on structural associations.

Second, the present study did not include data before July 2020 because risk perception was not assessed in the early months of the pandemic. This is unfortunate as the first months of the pandemic were indeed characterized by high levels of unpredictability of the COVID-19 virus, presumably causing even higher levels of risk than observed later in time. Also, additional aspects of risk perception could have been included, such as affective risk perception (e.g., the extent to which the risk makes people feel dread, [53]).

Third, common method variance (i.e., variance attributed to the measurement method rather than to the measured construct) must be considered a potential confound in the current research [54], because a large part of the constructs has been measured in the same survey on the same point in time. Also, for some of the variables, item characteristics were identical, as illustrated by the use of the same response scale for, e.g., motivation and adherence. Bias resulting from common method variance might not only impact the strength but also the direction of associations. Specifically, it might either inflate or deflate the observed relationships (i.e., leading to both Type I and Type II errors), depending on their association when method effects are controlled for [54]. This consideration does not apply to the objectively registered daily hospitalizations and infections, which yielded a rather differentiated pattern of associations with risk perception. Also, it is worth noting that we took a few design-related steps to mitigate this bias, such as emphasizing the anonymous and confidential nature of the survey and providing clear instructions to respond honestly and thoughtfully. Another step we took is that we randomized the order of items during data collection and we included only unique participants without repeated measures to reduce common method bias caused by sequence or memory. However, these measures may not fully eliminate the problem [54], and future research could consider additional measures to reduce common method variance by including, for instance, objective behavioral indicators (e.g., mobility data) as a different source of information or by using different response scales for self-reported assessments [54].

Finally, we did not account for other relevant covariates than sociodemographical variables to examine the robustness of the findings. Especially in the prediction of behavioral adherence, other factors might be relevant, such as the context-specificity of one’s behavior (e.g., role of strangers or friends who (fail to) comply with the measures [55]).

## Conclusion

Motivation played a key role in managing the COVID-19 pandemic, because health protective behaviors were required by policymakers. Although prior work indicated that volitional or autonomous motivation predicts better adherence of sanitary behaviors, the question which of the factors foster autonomous motivation received far less attention. The present study showed that especially perceived severity serves as critical, yet fluctuating driver of autonomous motivation. Using data being collected across 20 months of the COVID-19 pandemic in Belgium, risk perception steadily decreased, with a gradually widening discrepancy between vaccinated and unvaccinated persons. At the same time risk perception, especially the severity aspect, acted as a critical factor promoting internalization for both unvaccinated and vaccinated persons. This severity perception was itself more elevated on days when more people were hospitalized. The present findings have several implications for appropriate communication with the broader public to preserve optimal levels of motivation for and adherence to health protective behaviors.

### Electronic supplementary material

Below is the link to the electronic supplementary material.

Supplementary Material 1

## Data Availability

The R scripts to carry out the analyses are publicly available on Open Science Framework: https://osf.io/5cqhr/. Datasets are hosted in Zenodo (a public repository) and are available upon request and for replication purposes only (after contacting responsible researcher Joachim Waterschoot, Joachim.Waterschoot@ugent.be).

## Abbreviations

COVID-19: Coronavirus Disease 2019
SDT: Self-Determination Theory
(M)ANOVA: (Multivariate) Analysis of Variance
VIF: Variance Inflation Factor
MSEM: Multilevel Structural Equation Model
