# Supplementary material for: Risk perception as a motivational resource during the COVID-19 pandemic: the role of vaccination status and emerging variants
Source: BMC Public Health. 2024 Mar 6;24:731. doi: 10.1186/s12889-024-18020-z (PMC10918917; doi:10.1186/s12889-024-18020-z)
Supplement: Supplementary file 1 — Supplementary Material 1 [file 12889_2024_18020_MOESM1_ESM.docx]

**Supplementary materials**

Table S1. *Output of univariate analyses for categorical background variables.*

| **Variables** | **Variable** | | | ***F*-value** | ***p*-value** |  | ***η_p_^2^*** |
| --- | --- | --- | --- | --- | --- | --- | --- |
|  | ***Gender*** | |  |  |  |  |  |
|  | Male | Female |  |  |  |  |  |
| Perceived infection | 2.89 | 3.02 |  | 1,487.1 | <.001 | ^***^ | .00 |
| Perceived severity | 2.77 | 3.03 |  | 1,891.9 | <.001 | ^***^ | .02 |
| Autonomous motivation | 3.2 | 3.46 |  | 2,124.9 | <.001 | ^***^ | .01 |
| Adherence | 3.69 | 4.02 |  | 4,535.1 | <.001 | ^***^ | .03 |
|  |  |  |  |  |  |  |  |
|  | ***Comorbidity*** | | |  |  |  |  |
|  | **zero** | **one** | **more** |  |  |  |  |
| Perceived infection | 2.96 | 2.99 | 3.07 | 171.75 | <.001 | ^***^ | .00 |
| Perceived severity | 2.83 | 3.31 | 3.65 | 4,568.2 | <.001 | ^***^ | .06 |
| Autonomous motivation | 3.28 | 3.64 | 3.85 | 1,420.6 | <.001 | ^***^ | .02 |
| Adherence | 3.85 | 4.09 | 4.20 | 987.7 | <.001 | ^***^ | .01 |
|  |  |  |  |  |  |  |  |
|  | ***Education level*** | | |  |  |  |  |
|  | No secondary | Bachelor | Master |  |  |  |  |
| Perceived infection | 3.01 | 2.98 | 2.91 | 102.1 | <.001 | ^***^ | .00 |
| Perceived severity | 2.79 | 2.92 | 3.07 | 663.8 | <.001 | ^***^ | .00 |
| Autonomous motivation | 3.35 | 3.33 | 3.38 | 21.4 | <.001 | ^***^ | .00 |
| Adherence | 3.88 | 3.89 | 3.90 | 9.95 | <.001 | ^***^ | .00 |

*Figure S1.* Heatmap of calendar representing the number of participants in the current study.

*Note.* white cells refer to days when no data was collected
